# A Snapshot-Stacked Ensemble and Optimization Approach for Vehicle Breakdown Prediction

**DOI:** 10.3390/s23125621

**Published:** 2023-06-15

**Authors:** Reza Khoshkangini, Mohsen Tajgardan, Jens Lundström, Mahdi Rabbani, Daniel Tegnered

**Affiliations:** 1 Internet of Things and People Research Center (IoTap), Department of Computer Science and Media Technology, Malmö University, 211 19 Malmö, Sweden; 2 Center for Applied Intelligent Systems Research (CAISR), Halmstad University, 301 18 Halmstad, Sweden; jens.r.lundstrom@hh.se; 3 Faculty of Electrical and Computer Engineering, Qom University of Technology, Qom 1519-37195, Iran; tajgardan.m@qut.ac.ir; 4 Canadian Institute for Cybersecurity (CIC), University of New Brunswick (UNB), Fredericton, NB E3B 9W4, Canada; m.rabbani@unb.ca; 5 Volvo Group Connected Solutions, 417 56 Gothenburg, Sweden; daniel.tegnered@volvo.com

**Keywords:** breakdown prediction, optimization, deep neural networks, ensemble learning

## Abstract

Predicting breakdowns is becoming one of the main goals for vehicle manufacturers so as to better allocate resources, and to reduce costs and safety issues. At the core of the utilization of vehicle sensors is the fact that early detection of anomalies facilitates the prediction of potential breakdown issues, which, if otherwise undetected, could lead to breakdowns and warranty claims. However, the making of such predictions is too complex a challenge to solve using simple predictive models. The strength of heuristic optimization techniques in solving np-hard problems, and the recent success of ensemble approaches to various modeling problems, motivated us to investigate a hybrid optimization- and ensemble-based approach to tackle the complex task. In this study, we propose a snapshot-stacked ensemble deep neural network (SSED) approach to predict vehicle claims (in this study, we refer to a claim as being a breakdown or a fault) by considering vehicle operational life records. The approach includes three main modules: Data pre-processing, Dimensionality Reduction, and Ensemble Learning. The first module is developed to run a set of practices to integrate various sources of data, extract hidden information and segment the data into different time windows. In the second module, the most informative measurements to represent vehicle usage are selected through an adapted heuristic optimization approach. Finally, in the last module, the ensemble machine learning approach utilizes the selected measurements to map the vehicle usage to the breakdowns for the prediction. The proposed approach integrates, and uses, the following two sources of data, collected from thousands of heavy-duty trucks: Logged Vehicle Data (LVD) and Warranty Claim Data (WCD). The experimental results confirm the proposed system’s effectiveness in predicting vehicle breakdowns. By adapting the optimization and snapshot-stacked ensemble deep networks, we demonstrate how sensor data, in the form of vehicle usage history, contributes to claim predictions. The experimental evaluation of the system on other application domains also indicated the generality of the proposed approach.

## 1. Introduction

Machine Learning-based approaches for predictive modeling have become one of the main tools in the Predictive Maintenance (PdM) strategy of the automotive industry, to improve the overall maintenance and reliability of operations, and to reduce costs and safety issues [1]. Such predictive models attempt to model and predict the breakdown of an asset (e.g., truck, bus, or car) which, if it occurred within the agreed warranty time period, could lead to a warranty claim. This set of systems is precious software technology that minimizes unexpected down-time which, in turn, improves safety and asset reliability. PdM is highly valuable for reducing costs as it means maintenance is performed only when necessary. Based on the figures reported in [2], maintenance is one of the leading vehicle operational expenses, accounting for around 59% of overall costs. Furthermore, under the PdM umbrella, claim or breakdown prediction of upcoming down-time significantly decreases the risk of human life being jeopardized in, for example, heavy-duty vehicles (in this study, the data source for the target labels is a warranty claim database and, therefore, we interchangeably use down-time, claim, breakdown and fault, such that they all point to the concept of a component defect leading to a claim in the dataset).

Heavy-duty vehicles are complex systems, with numerous possible specifications, operations and driver behaviors, wherein component down-times can originate from multiple sub-components failing for various causes. The correct prediction of breakdowns is critical and essential because failing to precisely recognize such breakdowns leads to increased maintenance costs, increased customer safety risks, decreased customer satisfaction, and lowered brand value. All of these factors add up to a significant loss for companies. Thus, correctly predicting faults that could cause customer or company loss has become an important goal in today’s modern industrial strategy. Under this vision, many studies have investigated the prediction of warranty claims or component breakdowns, reliability, and degradation in the context of the vehicle industry, utilizing machine learning and statistical approaches, such as deep neural networks, recurrent neural networks, support vector machines or stochastic approaches [3,4,5]. Diagnostic models were developed, based on the data collected from machines (e.g, industrial machines, turbines, vehicles, trains, etc.) to estimate the performance and condition of a component. Many of the studies utilized stochastic approaches to estimate component failures [6,7]. Many studies have focused on estimating the remaining useful life (RUL) of a component, utilizing optimization or neural networks algorithms [7,8,9,10]. For example, Yang et al. [11] studied claim forecasting, where they focused on product usage rate and introduced two predictive models. In this study, the authors considered Weibull’s distributed time assumption to foretell the number of breakdowns. More recently, an interesting work by Chehade et al. [12] studied the problem of data maturation in predicting warranty claims. They utilized Gaussian Mixture distribution of claim patterns from similar vehicles to forecast vehicle warranty claims as early as possible [12].

From the literature, we observed that considerable progress has been made in the context of predicting upcoming breakdowns in the automotive industry by adopting machine learning and statistical solutions. We also noticed that most of these combine and use the approaches with the age and lifetime of a particular component to estimate the component’s breakdown. However, several recent studies in such areas [13,14] partially use vehicle usage to predict the need for maintenance to avoid breakdowns. Complexity, both in terms of formulating the problem and the high dimensionality of the usage data, make the task challenging, requiring further investigation.

Although the works mentioned vary widely in terms of the application context, problem to solve, and technique developed to tackle the challenge, machine learning has proved to be successful, particularly in the automotive industry [15]. However, there is still room for improvement (in terms of prediction accuracy and explainability) in existing claim prognostic systems. We believe that such forecasting operations can be improved by incorporating AI and advanced deep neural network approaches.

This motivated us to develop an ensemble solution taking advantage of hundreds of sensor signals to predict breakdowns (which often lead to warranty claims, the source of our target variable). However, the challenge of high-dimensional data needs to be dealt with. Thus, in this study, we propose an ensemble method to extract and select the best representation of the data–the most informative features–to build the predictive models for warranty claim prediction (WCP). The approach includes three modules. In the first module, that of “Data pre-processing”. we developed a set of practices to integrate various sources of data, to extract relevant information and to segment the data into various time windows. In the second module, that of “Dimensionality Reduction”, we designed an optimization procedure, using a Genetic Algorithm (GA) coupled with Elastic Net, to translate high-dimensional data into a representation with lower dimensionality without compromising prediction performance. GA is a method inspired by the evolutionary process and is used for solving constrained or unconstrained optimization problems [16]. The method runs several GA operators that frequently change individuals to reach the best solutions through several generations.

In the third module, that of “Snapshot-stacked Ensemble”, which contains two phases, we developed an ensemble deep neural network to use the measurements (predictors, measurements, and features all refer to the same concept of sensor data) to map vehicle usage to claims. This module was developed by means of a Snapshot-stacked Ensemble method to increase the performance of the predictive model. In this fashion, several training learners are built from different perspectives to solve a complex claim prediction using vehicle usage. This is achieved by horizontally aggregating the outputs of several snapshot models, utilizing a Cyclic Cosine Annealing Schedule (CCAS) [17] concerning the principle of diversity, to improve predictive model performance. Indeed, each snapshot refers to a separate deep neural network, generated over one training process and saved, which is then used to build an ensemble model. These snapshots result in meta-learning that is capable of learning from various models (deep neural networks) to increase the accuracy of the claim prediction. In other words, we exploit the outcome of each snapshot to build an ensemble of networks for better prediction.

To the best of our knowledge, this study is the first to investigate the performance of ensemble optimization and snapshot-stacked deep neural networks to translate higher dimensional data into a lower dimension to be utilized for claim prediction. Earlier fault estimation was done through manual inspection and statistical approaches. Applying artificial intelligence techniques on sensor data automates fault detection, requiring less effort and achieving more accuracy in a timely fashion [18].

The following research questions (RQs) further elaborate the investigative objectives of our proposed approach:

- **RQ1—Measurement selection:** How could vehicle usage best be extracted for use in claim prediction, and to what extent could the extracted features contribute to the predictive model?
- **RQ2—Claim prediction, based on vehicle usage:** To what extent could ensemble deep neural networks contribute to, and improve, claim prediction in the automotive industry?

Our study aimed to address claim prediction by mapping vehicle usage to future breakdowns. First, we concentrated on selecting a subset of features that would best represent vehicle usage. Since heuristic and optimization solutions have demonstrated their capabilities to tackle np-hard problems in various domains [19,20], we hypothesized that the genetic method would be able to address this issue. Thus, to answer RQ1 we adapted the evolutionary approach to feature selection so as to select the sub-optimal features. The evaluation was performed over several generations and compared with other standard deep neural network and ensemble approaches. The contribution of the extracted features to the predictive model was evaluated by means of an A/B test, guided by the aforementioned RQ1. We report on the results of that optimization and A/B test, to show how the selected and extracted features positively affect the performance of the model. Second, we maintained that the optimization could perform better by coupling it with an ensemble learning approach. Hence, to answer RQ2, we developed a Snapshot-stacked ensemble learning system through two phases of generating multiple diverse models, which were used to build meta-learning. The meta-learning was then utilized for the final claim or breakdown prediction. The reported figures confirm that this development improves claim prediction performance compared with other ML approaches. The integration of these two methods in predictive maintenance strategy, which our research outlines, constitutes this paper’s main contribution. The following are our contributions in this study:

- Whereas previous studies mostly used limited features in their prognostic systems, we used hundreds of real sensors’ data (LVD) from heavy-duty trucks to model and map vehicle usage to component breakdown.
- Taking into consideration the high dimensionality of sensor data, an optimization approach was introduced to extract the best representation of the data characterizing vehicle usage in order to build an accurate predictive model for warranty claim forecasting.
- Taking into consideration the low classification accuracy of claim prediction using vehicle usage, a snapshot-stacked ensemble deep learning approach is proposed in this paper. Compared with existing algorithms, our method effectively improves classification performance.

The rest of the paper is organized as follows. Section 2 presents related studies. In Section 3, we describe the data used. In Section 4 we explain the proposed approach. Section 5 covers experimental evaluation and results, followed by a discussion and summary of the work in Section 6.

## 2. Related Works

Predictive maintenance techniques have previously been investigated and developed to examine and monitor the condition of tools and equipment to estimate when maintenance needs to be performed. In this regard, identifying component failures proffers opportunities for companies to take preemptive action, in the form of predictive maintenance, to minimize breakdowns and prevent more significant issues in the long term. This mitigates safety risks, lowers costs and even supports sustainable production in smart cities [21,22]. Many attempts have been made [23,24,25] to develop various statistical and machine learning solutions to predict component failures in a wide range of applications such as the following: manufacturing [26,27], automotive [28,29,30,31], and energy [32]. Prognostic models have been based on data collected from machines, social networks, and other sources to estimate the performance and condition of components or the number of upcoming warranty claims [33]. These models widely used statistical approaches to estimate component failures [6,7,34,35]. For example, in the early 1990s, in [34], a log-linear Poisson model was utilized to build a forecasting system based on the dates of warranty claims to predict failures. Later, Fredette et al. [5] developed a mixed Non-homogeneous Poisson Process (NHPP) to tackle a similar problem, that of component down-time prediction. An interesting remaining useful life (RUL) forecasting approach was introduced in [7], in which the Wiener process [36] with drift was utilized to model degradation in component sensors. Recently, in [33], social media data was used to improve performance in daily warranty claim prediction. To tackle the challenge of warranty data maturation, an intriguing study was developed by Chehade et al. [12]. This investigation used warranty claim data to devise a Gaussian mixture distribution of claim patterns at current and future maturation levels. They took advantage of Bayesian theories to assess the conditional posterior distribution of warranty claims at future maturation based on the available data at the current maturation level.

To the best of our knowledge, the larger part of studies focusing on forecasting problems widely use neural networks to estimate the RUL of components and the state of complex machines [37]. One example is in the context of energy production [38], wherein a self-evolving maintenance scheduler technique was proposed to detect damage in the gearbox bearings of wind turbines. The technique uses an ANN to establish a condition monitoring system (CMS) based on supervisory control and acquisition data. To tackle a similar problem, in [39] a Sparse Autoencoder-based Deep Neural Network (SAE–DNN) approach was developed to detect bearing breakdowns in multi-component systems.

A typical optimization approach was introduced in [9], using Relevance Vector Machine, with the support of ant colony optimization (ACO), to detect gearbox fault detection.

To tackle a similar problem, a neural network-based forecasting system was introduced in [40], based on a dynamic Cuckoo search optimization algorithm. Such forecasting is crucial, since it can help manufacturers lower maintenance costs, increase customer satisfaction, and safety, etc.

Lifetime distribution estimation as a practical solution has gone a long way in component fault estimation. For instance, a probabilistic model was developed by Singpurwalla et al. in [41], wherein time, and time-dependent quantity, were utilized to build a forecasting model. Later, Kaminskiy et al. [42] proposed a component failure prediction approach, based on piece-wise application of the Weibull and exponential distributions, to predict intrinsic failure period and early failure period. Similarly, in [43] two variables of age and mileage of the vehicle were considered to estimate the mean cumulative number of breakdowns.

**Figure 1:**
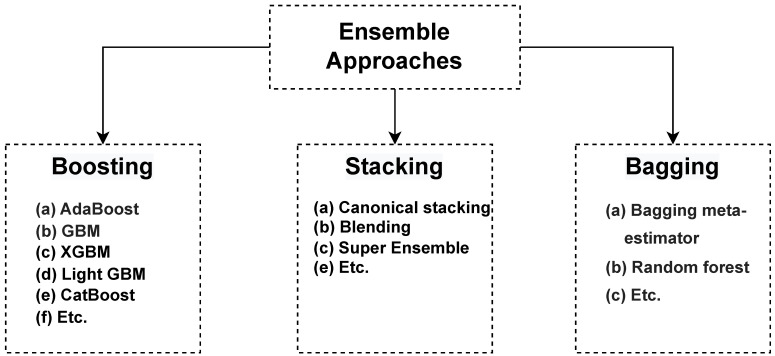
Taxonomy of ensemble approaches.

Among the vast numbers of approaches introduced and developed for various prediction tasks in many applications, ensemble techniques have been quite successful. Ensemble machine learning approaches are techniques that integrate several base ML models to provide improved performance [44,45,46,47,48]. As illustrated in Figure 1, these techniques can be divided into three categories: Stacking, Bagging, and Boosting. The Stacking, or Stacked generalization, technique [49] uses and combines predictions from multiple predictive models to build a new model on the training data. Then the model is utilized for the final prediction of the test data. Bagging is a technique that seeks a mixed group of ensemble predictive models built by various parts of the training data [50]. Then, statistical approaches, such as averaging or voting, are used to combine prediction results from members. Boosting is another ensemble approach that creates a strong predictive model from several weak classifiers [51]. In this approach, the training data is used to build a predictive model. The second model then tries to correct the mistakes from the first classifier. This process continue until the maximum number of predictive models is reached or the perfect prediction acquired.

Researchers working on applications from different sectors also took advantage of ensemble methods [52]. In the context of claim and fault detection, we observed interesting studies in different sectors [53,54]. For example, a bagging ensemble-based approach was developed in [55] to forecast faults in a single-shaft industrial gas turbine. In this approach, the fault prognosis was based on weighted voting to improve the robustness of the fault detection technique. To tackle the same problem in a similar domain, in [56] a deep neural network ensemble approach was developed and applied to a wind turbine data series. This work trained the network offline based on a training set of the imagination matrix that transformed the data series through segmentation technology. Then, the network was retrained by transferring learning, based on the renewed training set, to achieve enough knowledge to forecast the fault. In [57], an ensemble approach was introduced to predict the degradation of track geometry, wherein different models were developed by means of regression, deterioration, and classification. In the context of an electrical system, ref. [54] introduced an arc series ensemble fault detection approach, wherein they developed a boosting converter CPL and a buck convertor constant power load (CPL) to study different arc fault behaviors.

All in all, two main limitations can be observed in the related works presented: (1) most are limited to models based on only a few input parameters to predict warranty claims; (2) lack in use of complex real-world operational data from thousands of deployed products, which come with the challenges of noisy and missing data. Our study addresses these issues by deploying a complex ensemble-based system on high-dimensional data collected from thousands of heavy-duty trucks.

To our knowledge, the combination of evolutionary technique and ensemble deep neural network as an approach to increase warranty claim prediction accuracy, is novel. Our approach addresses the fault detection problem in the automotive industry, which is generic and applicable to a variety of fault and claim predictions in different sectors. Borrowing from the taxonomy in [56], our warranty claim prediction can be characterized as a stacked ensemble approach, since it takes into consideration the prediction of several diverse snapshot models to build meta-learning, and then the model is used for a final prediction. In the remainder of this paper, we describe the data, our proposed solution and the evaluation of its potential, both for warranty claim data and datasets from other applications.

## 3. Data Presentation

In this section we present the following two data sets which were used to test the proposed forecasting method: Logged Vehicle Data (LVD), including specifications, and usage, of vehicles aggregated in a cumulative fashion; Warranty Claim data (WCD), consisting of claim information reported over the vehicles’ life-times during their warranty periods (24 months).

### 3.1. Logged Vehicle Data

**Figure 2:**
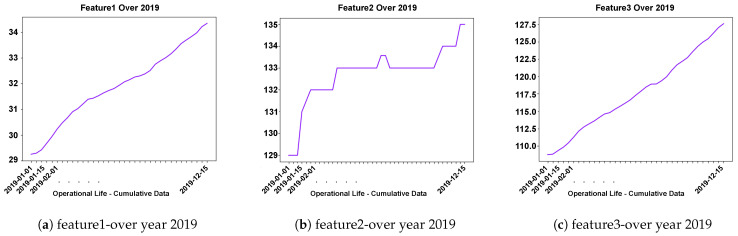
Illustration of the vehicle sensor data logged as scalar features. The scalars indicate the vehicle usage over one year in 2019. As is clear, the parameter was cumulative and monotonous–in feature1 and feature3–and increased over the year.

The LVD used in this study were collected from commercial trucks over a four-year period, from 2016 to 2019. The LVD consisted of the aggregated usage information for a fleet of heavy-duty trucks operating in Europe. The parameters were collected using telematics, as well as through manual inspection when a vehicle visited an authorized workshop for repairs and service. In general, two types of parameters are logged in the LVD. The first type describes the configuration of the vehicles, e.g., engine type, gearbox specification, or type of pumps. The second type logs the usage of the vehicle during its operation. This data is logged in histogram and scalar formats, continuously aggregated and contains a number of different parameters, such as fuel consumption, compressor usage, gear usage, cargo load, engine ambient temperature vs. ambient pressure log, coolant temperature vs. oil temperature log, etc. An example of cumulative scalar features and its structure over one year are illustrated in Figure 2.

### 3.2. Warranty Claim Data

Claim data contains information regarding a vehicle’s warranty claims logged during its operation, collected by OEM authorized workshops in Europe. In particular, the claim database stores information about which part or component of a vehicle was repaired or changed (due to a failure) along with the date. The parts and components are identified by the normalized identification codes using four different levels of detail. This claim dataset contains various parameters, such as names of components, codes, descriptions, and dates, etc.

## 4. Proposed Approach

### 4.1. Problem Formulation

This section represents the formulations determined to tackle the claim forecasting problem. Our approach considers the WCD and LVD as the system’s inputs and employs optimization and ensemble-based deep neural networks. This study attempted to predict, one month beforehand, defects related to the power train in heavy-duty vehicles, during their warranty period of 24 months from when they started operating in service. Thus, our intention in this claim prediction investigation could be divided into two problem formulations, as follow:

- First, we formulated the process of dimensionality reduction as an optimization task, and developed an optimization approach to extract a better set of features characterizing vehicle usage over time. The approach was carried out in a time-series fashion based on the hypothesis that vehicle behavior changes over time, so different predictors might have different impacts on claims/failures and, consequently, on performances of predictive models.
- Second, we investigated the design of an ensemble machine learning approach, formulating the task as a classification to predict the claim/defect related to a power train. Given the selected predictors from the optimization step, the ensemble approach mapped the usage to the upcoming claims one month ahead during the warranty period.

**Figure 3:**
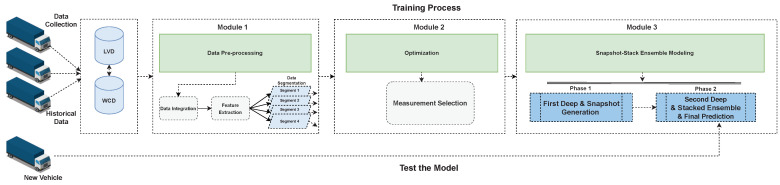
The conceptual view of the proposed breakdown prediction approach.

Figure 3 shows the high-level structure of the proposed claim prediction approach for the automotive industry. It contains three main modules: Data pre-processing, Feature Selection Optimization and Ensemble Modeling. The data pre-processing module consists of three processes: data integration, feature extraction, and data segmentation. The optimization module is then employed to choose the measurements in a time-series fashion. Here, the best representation of data in each segment is transferred to the ensemble modeling module. Subsequently, the ensemble method builds the predictive model over two phases to forecast warranty claims, given the vehicle usage. These modules and sub-modules are described as follow:

### 4.2. Module 1: Data Pre-Processing

Data preparation is an integral part of any machine learning problem and our fault diagnosis task is not an exception. In this section, we describe how we prepared the data to use in our optimization process.

#### 4.2.1. Data Integration

This module aimed to merge the LVD and WCD to create an integrated dossier with the usage and failure information. We combined two data sets based on the vehicle’s “Chassis id”, “Date of readout, which is logged”, and “Date of failure report”. To merge these two datasets, we borrowed the method that we used in our previous study [18], where we took advantage of a Volvo expert’s knowledge to determine a time window of one month as an interval where the symptoms of failures are most likely to be visible. Therefore, the prediction horizon was set to 30 days for the developed model. In this way, the merged data-set included a new attribute named “Target” as the target feature *T*. This parameter has a value of 1 for a given sample ($r_{i}$) if, and only if, failure of a specific component of interest is reported. Therefore, each vehicle may have multiple failures (for the same component) during its operational life. However, only one failure (for the same component) is considered during the desired time window. More formally, each time-window/time span is assigned a binary label according to Equation (1), where tw refers to the length of the time window that has the highest impact on failures in trucks and where τ is the current time (end of time window). Note: tw is determined based on a Volvo expert’s knowledge, which differs from one type of failure to another. Thus, in this study, tw was set to one month which was considered large enough to overcome the effects of choosing a window that is too small, e.g., having a prediction horizon that is too tight or not being able to cover the whole chain of events leading to a failure. If a shorter time window would have been chosen, the target feature would have made more sense to be continuous, due to gradual transitioning into the chain of events.

(1) $$T_{r_{i}}=\left\{\begin{array}{c} 1\;\;\mathrm{if}\;a\;\mathrm{fault}\;\mathrm{happened}\;\mathrm{in}\;\;[\tau -tw,\tau ]\\ 0\;\;\mathrm{if}\;\mathrm{no}\;\mathrm{fault}\;\mathrm{happened}\;\mathrm{in}\;\;[\tau -tw,\tau ] \end{array}\right.$$

#### 4.2.2. Feature Extraction

This sub-module computes the differences between consecutive measurements of each sensor signal accumulator and quantizes the features. Indeed, this process was used due to the accumulative nature of data. It is essential to extract the actual usage in each timestamp, so that an abnormality estimating a breakdown in the near future might be indicated.

Equation (2) shows how a new feature (from each original feature) is calculated:

(2) $$f_{i_{new}}=f_{i_{r_{i+1}}}-f_{i_{r_{i}}}$$

(3) $$Quarterlies=\left\{\begin{array}{c} Q1\;\;(n\;+\;1)/4\\ Q2\;\;(n\;+\;1)/2\;\\ Q3\;\;(3(n\;+\;1)/4)) \end{array}\right.$$

(4) $$Cat=\left\{\begin{array}{c} low\;\;\mathrm{if}\;f_{i_{new}}<Q1\;\\ medium\;\;\mathrm{if}\;Q1\;<f_{i_{new}}<Q3\;\\ high\;\;\mathrm{if}\;f_{i_{new}}>Q3\;\;\; \end{array}\right.$$

where $f_{i_{new}}$ denotes the extracted feature, and $r_{i}$ and $r_{i+1}$ represent the readouts that are collected over the time and *n* points to the number of samples or readouts. Basically, in the feature extraction module, we calculate the *delta* between subsequent readouts, and exploit them as new features in training the models. Indeed, these extracted features demonstrate the actual usage (and their changes) of vehicles in a bi-weekly fashion. The anomaly behavior of the vehicle hidden in the data is revealed from these changes, which might indicate potential faults in the near future. In addition, information related to each season (spring, summer, autumn and winter) is added as a new feature in the data.

**Figure 4:**
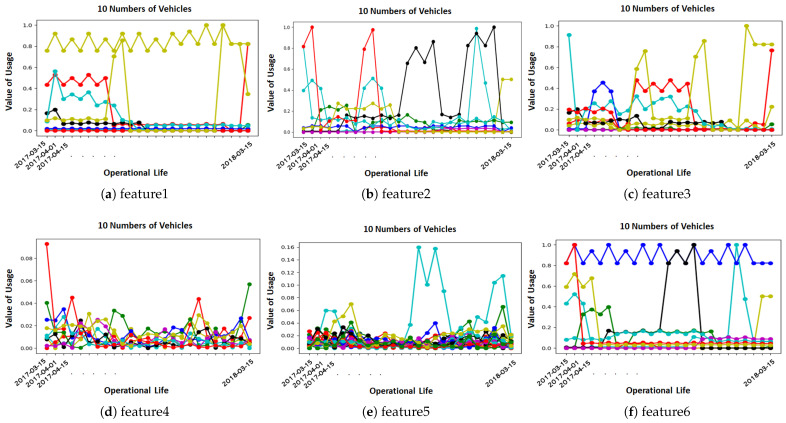
Illustration of the extracted features ($f_{i_{new}}$) from ten vehicles. The subplots (**a**–**f**) show significant (formulated in Equation (2)) and gradual changes bi-weekly in each parameter over one year. This means the original LVD data were logged every two weeks and collected by the authorized OEM workshops when the vehicles visited the workshops anywhere in Europe.

Figure 4 depicts only the usage change of 10 vehicles considering 8 new features ($f_{i_{new}}$)–out of more than 600 features in total. Furthermore, we quantified the level of usage change $f_{i_{new}}$ into low, medium, and high changes. This quantization was done with the support of expert knowledge from Volvo engineers in the field. Equations (3) and (4) explain how the quartiles, and accordingly the level of changes in vehicle usage, are defined. In this way, we assume that substantial change (reduction or raise $f_{i_{new}}>Q3$) in the usage of vehicles leads to performance degradation and component failure in the future.

To show how the changes might relate to failures, we grouped the vehicles into two categories of Healthy and Unhealthy vehicles. A healthy vehicle is one that has no faults during its operational life, while an unhealthy vehicle has at least one reported claim in its history.

**Figure 5:**
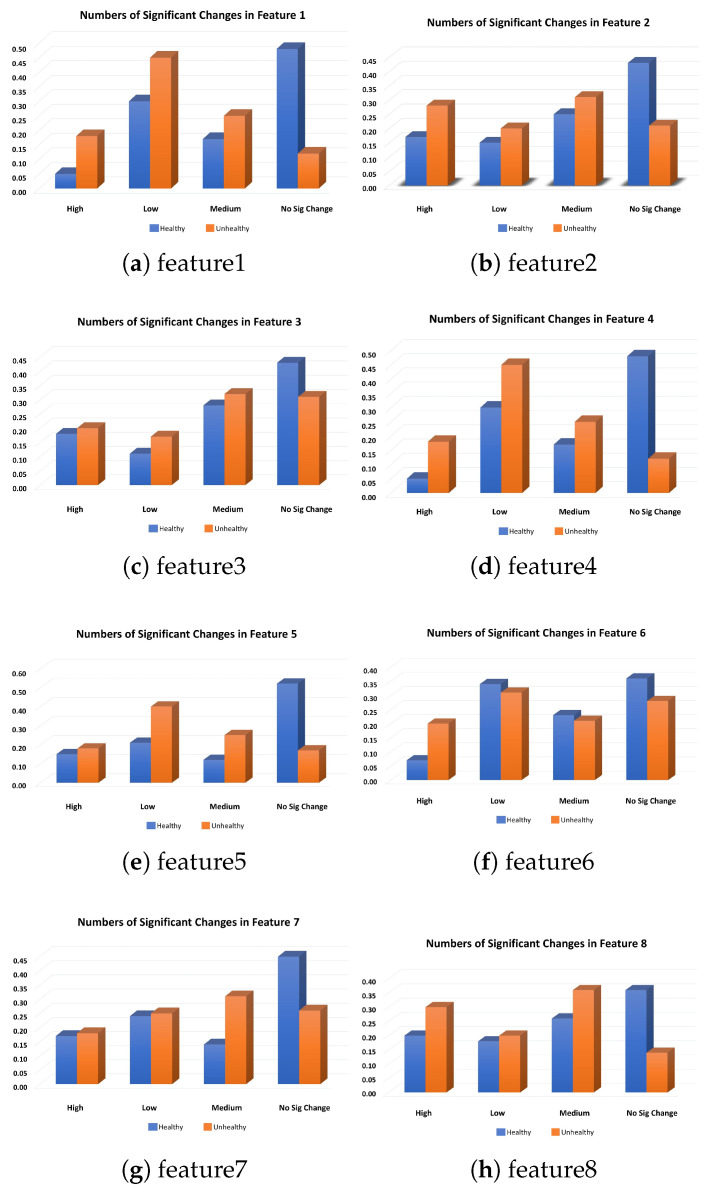
Illustration of significant usage changes in healthy and unhealthy vehicles. These subplots show how the significant usage changed in eight different features (extracted features), related to the health of the vehicles. Note: we used more than 600 features in our study, and these were just eight features we randomly selected for the purpose of plotting.

Figure 5 demonstrates how the changes are related to the groups of vehicles, where the *y*-axis explains the relative frequency of usage change in four different categories. The vehicles with high, medium and low or no numbers of significant changes are shown on the *x*-axis. It can be observed from the usage pattern, plotted through the sub-figures, that the proportion of significant changes in unhealthy vehicles was higher than in healthy vehicles during their life histories (except one depicted in Figure 4f). Likewise, the proportion of healthy vehicles was more than that of unhealthy vehicles once we considered no-changes to evaluate the association between them. Through this experiment, we concluded that healthy vehicles had less usage deviation than unhealthy vehicles. Thus, exploiting this information contributed to building a better predictive model to result in more accurate predictions.

#### 4.2.3. Data Segmentation

The segmentation was conducted by having hypotheses in which vehicle demeanor differs from context to context, and the context has a certain impact on the vehicle’s performance. In the domain of the automotive industry, context can be characterized as location, application domain, time, specific road conditions, etc. Furthermore, this segmentation might aid to increase the performance of the predictive model. Thus, in this study, with this low-resolution data, the operational time of the vehicles over different seasons was considered to segment the data. Basically, we partitioned the whole time-series data into four segments representing the four seasons in a year.

This time-series data set contained 337,573 samples with 375 original features (parameters/measurements), characterizing the usage style of 2412 unique vehicles over four years of operation. In addition, information related to each season was added as a new feature in the data. In this way, in each segment of time we defined three months as a season over a year, and each time segment contained the vehicles which operated in that period of time in different years (regardless of their ages or production months). Since vehicles have different ages and operational patterns over the year, each segment contained various numbers of samples/readouts as follows: Segment1 contained 86,768 readouts; Segment2 included 90,709 readouts; Segment3 held 61,364 readouts, and, finally, Segment4 contained 98,732 readouts.

Bearing in mind the assumption raised in the integration section (Section 4.2.1), to label the LVD (one-month operation may have the highest impact on component down-times), we took account a very rare case of failure on the first day of each season, so each segment also contained one-month operation from the previous season. This allowed the model to map the usage to a failure if the failure happened on the first day of the season.

### 4.3. Module 2: Optimization: Measurement Selection

This section describes the module developed to conduct the measurement selection process. We formulated this process as an optimization task, wherein we aimed to find the best representation of vehicle usage (a subset of measurements/features) to increase the predictive model’s performance in forecasting component failure. In other words, our objective was to discover the optimal subset of measurements that could characterize vehicle usage and map these to failures. Thus, to this end, we constructed this module by modifying the original Genetic Optimization [58], which was successfully applied in a wide range of applications [59,60], just to cite a few. As the main contribution in this module, we utilized the Elastic Net [61] technique, by adding simple penalties (a combination of L1 and L2 regularization) into the optimization techniques (we call this ELSGA), which acted as a generation component to initiate a decent population in the first step for the optimization process. Although, data dimensionality reduction approaches, such as PCA [61,62] or LDA [63], could be implemented for this problem, in order to maintain interpretability, using such methods was avoided in this dimensionality reduction step. The optimization module itself consists of three main sub-modules: Initialization component (IC), Evaluation component (Objective function) and Criterion. These sub-modules are described as follows:

#### 4.3.1. Initialization Component (IC)

Given the desired segment ($Seg_{i}$) from the segmentation module, IC initiates the first generation of the population (rather than randomly select the first generation of features, which is the fundamental process in the GA algorithm, we aimed to start the optimization process with better population of the measurements) using a convex integration of lasso [64] and ridge [65] methods called Elastic Net (LS) [66]. We took advantage of the LS capability, that uses L1 and L2 penalties to shrink and select features, to generate the first population of predictors. Let us suppose we have $R=(r_{1},\ldots ,r_{n})$ readouts in the given time series $Seg_{i}$ data-set with $F=(f_{1},\ldots ,f_{m})$ features/predictors (measurements), and $T=(t_{1},\ldots ,t_{n})$ as the target variables.The value $a_{i,j}$ refers to the value of feature $f_{i}$ in readout $r_{i}$.

$$R=\left(\begin{array}{cccc} \; & f_{1} & \cdots & f_{m}\\ r_{1}= & a_{1,1} & \cdots & a_{1,m}\\ r_{2}= & a_{2,2} & \cdots & a_{2,m}\\ \vdots & \vdots & \ddots & \vdots \\ r_{n}= & a_{n,2} & \cdots & a_{n,m} \end{array}\right)\times T=\left(\begin{array}{c} T\\ t_{1}\\ t_{2}\\ \vdots \\ t_{n} \end{array}\right)$$

Thus, *R* and *T* are our model matrix, and prediction of target variable $\hat{t}$ can be described as:

(5) $$\hat{t}=\sum_{j=1}^{m}\beta_{i}f_{j}=\beta^{T}F$$

where the input function is the dot product of the predictors and coefficients β:

(6) ∑j=1m=β1f1+β2f12+⋯+βmfm

Thus, in our initialization process, the task is defined as a classification problem, so β can be calculated by Equation (7):

(7) $$Elastic\left(\hat{\beta }\right)=\left(tf\right)+\lambda \left[\left(1-\alpha \right){∥\beta ∥}^{2}+\alpha {∥\beta ∥}_{1}\right]$$

(8) $$\left(tf\right)=argmin\sum_{k=1}^{n}\left(y_{k}P_{k}^{T}\beta \right)$$

(9) $${∥\beta ∥}_{1}=\sum_{j=1}^{m}\left|\beta_{m}\right|$$

(10) $${∥\beta ∥}^{2}=\sum_{j=1}^{m}\beta_{m}^{2}$$

where is a margin-based loss function and y∈{0,1}. Using ${∥\beta ∥}_{1}$ and ${∥\beta ∥}^{2}$, which are the norm $l_{1}$ and euclidean norm, we measure the largeness of the coefficient.

λ and α are the two parameters to tune. α controls the integration of the two penalties and λ manages the amount of penalization, where α can take values between [0–1], and λ could be a positive number.

There are two alternatives for the LS: LS turns to ridge method once α gets close to 0, while LS serves as lasso when α reaches 1. Thus, to find the best α and l1, we tuned these parameters through walk-forward cross-validation by the learning process. This means we trained different models exploiting various values in an optimization manner to find the best setting. In this way, the algorithm overcame the drawbacks of the Lasso and Ridge functions [67] to select the best predictors. Thus, in IC we used an agent to perform Equation (11) to initialize the first population to be delivered to the Evaluation component and kept generating such decent populations to inject from the GA sector over the process.

(11) $$IC=\left\{\begin{array}{c} Rand(\frac{1}{2}*LS\left(seg_{x_{j_{f_{1,\ldots ,m}}}}^{i}\right),pop)\\ i=1,\ldots ,m\\ j=1,\ldots ,n\\ p=1,\ldots ,m\\ pop=\left[6,8,10,12,14\right] \end{array}\right.$$

where Rand() is a random selection of decent initiated predictors by $LS(seg_{i})$ and Rand() is designed to keep the diversity within the decent selected predictors. The value pop denotes the size of the population that should be initialized at the first generation. The value of pop was tuned with the above five numbers to find the optimal number for the population. The reasons for developing such an initialization component, rather than random generation, are as follows: (a) we hypothesized that good parents most likely generate better children than bad (or random) parents. This means randomly initializing the parent may lead to non-satisfying predictive performance in the fitness function, ignoring the cross-over step, and, accordingly, terminating the Genetic process; (b) in any optimization process, it is crucial to reach the optimal solution in the early stages (generations), particularly when the system needs to deal with a huge amount of streaming data. Thus, the random selection may lead to post-bonding to the late generations; (c) the nature of the GA approach is based on random generation and selection of a population to obtain the best solution; therefore, the approach does not concern the correlation between the features and the predictive performance. There might be highly correlated features that can contribute together to performance. Hence, the design and integration of IC support the GA operators by injecting such information to generate a better population over the optimization process. For this reason, we employed LS in this initialization component since there might be highly correlated predictors in the high dimensional data; thus, LS automatically included all the highly related predictors in the population if one of them was selected. However, it needs to be remarked that there might be cases among these highly related features wherein only one is sufficient and contributes to the predictive performance, and so GA solves such an issue.To summarize, this design with LS and GA allows the inclusion of the many correlations expected, since features coming from sensors measure correlated processes. However, the GA-enabled part of the algorithm tries to select the best subset out of such correlations and does not necessarily keep all of them. The ensemble of these two algorithms overcomes the raised concerns, particularly as the automotive sector deals with high-dimensional streaming data over time. This enables the system to decrease dimensionality and, at the same time, increase the performance of the predictive model.

#### 4.3.2. Evaluation Component (Fitness Function)

At the core of this component, and of the GA process, a decision tree-based ensemble method [68] (XGBoost) was used as a classifier to evaluate the importance of the individuals and their impacts on the predictive model. This objective function of claim prediction L(θ), can be expressed as follows [68]:

$$L\left(\theta \right)=y_{i}log\left(logistic\left(y_{i}\left({\hat{y}}_{i}^{t-1}+f_{t}\left(r_{i}\right)\right)\right)+\left(1-y_{i}\right)log\left(1-logistic\left({\hat{y}}_{i}^{t-1}\right)+f_{t}\left(r_{i}\right)\right)\right)$$

where *L* is the log-likelihood function, and, thus, $logistic(y_{i}\left({\hat{y}}_{i}^{t-1})\right)$ is the probability. $f_{t}$ denotes the *t*th tree, and $y^{t-1}$ shows the prediction of $r_{i}$ sample, at t−1 iteration. In this experiment, we used the logistic function, as our problem was formulated as a binary classification. Indeed, the merit of each population represented as solutions are evaluated as fitness value, so the best chromosomes are ranked and selected for the next generation. We formulated our fitness function as an optimization, where individuals with higher performance are considered for the next generation. In a parameterized fashion, the four top best populations were selected, in the selection process to be given to GA operators for cross-over and to generate a new solution (offspring).

A typical crossover method, called Partially Mapped (PMX) [69], was used in this optimization problem. The main idea behind this decision was to support the GA process by having a better convergence rate and diversity and to reduce stagnation during the optimization process [70].

#### 4.3.3. Criterion Component

Three criteria were incorporated into a composite optimization approach as follows: *(1) poor fitness value ($Tr_{1})$, (2) maximum fitness value ($Tr_{2}$),* and *(3) maximum generation ($Tr_{3}$)*. The first criterion talks about the poorness of generated predictors by the IC component at the first generation. The IC is called if, and only if, the fitness value could not pass the minimum threshold.

(12) $$Cr=\left\{\begin{array}{c} Tr_{1}\;is\;met\;\;\mathrm{if}\;f(x)\;\geq \;0.5,otherwise\;\;call\;\;IC\\ Tr_{2}\;is\;met\;\;\mathrm{if}\;f(x)\;\geq \;0.95,then\;\;terminate\;\;GA\\ Tr_{3}\;is\;met\;\;\mathrm{if}\;\mathrm{itr}\;=\;\max\;\mathrm{gen},then\;\;terminate\;\;GA \end{array}\right.$$

This criterion was established to ensure the first population has the potential for further generation during the optimization process. The second criterion indicates that the fitness output has been sufficiently reached for the optimization process to be terminated once it is met. As the third proxy $Tr_{3}$, we defined a maximum generation. Taking into account the first criterion $Tr_{1}$, once one of $Tr_{2}$ or $Tr_{3}$ is met, the selected operators/features are injected into the ensemble modeling (in the third module) to build a complex and general predictive model for the final claim forecasting.

### 4.4. Module 3: Snapshot-Stacked Ensemble Modeling (Network Construction and Training)

In this section, we describe the third module of the proposed approach that contains two main phases. In the first phase, we constructed an ANN, which was used to generate several snapshot models from a single training process. Then, we took those snapshots (models) for validation, and, in the second phase, the prediction output (class probabilities) of each snapshot model was added to the dataset for final training and prediction. Indeed, the second phase acts as a meta-learning process, where it attempts to learn from the output of the snapshots to reduce the classification errors obtained from the first network.

#### 4.4.1. Snapshot Ensemble on Vehicle Usage

**Figure 6:**
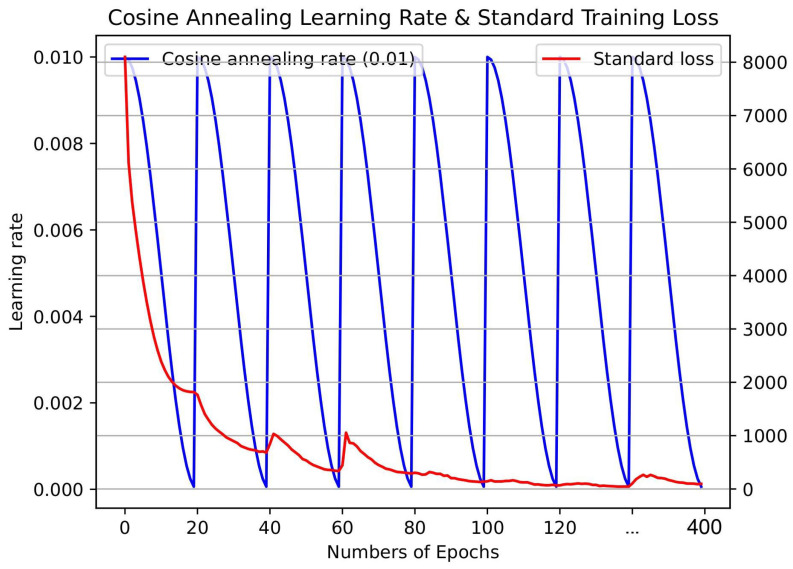
Visualization of the standard loss (red) and cosine annealing learning and cycles (blue). In this practice, N = 20 snapshot models were generated and saved to be used in the first and the second phases of the networks.

The ensemble technique and its modeling is a process in which several models are generated to forecast the outcome. This can be done by utilizing different predictive models or different training data sets. The ensemble model then combines each model’s output to obtain the final prediction for the test data. The method highly supports the network to increase the predictive model’s performance compared to the individual model by decreasing the generalization error. Since training multiple deep networks is computationally expensive, we utilized Snapshot Ensemble to train multiple models by a single training process [71]. We utilized this approach, which we introduced in [71], and adapted and integrated it into the optimization technique for the breakdown prediction. However, this technique has the challenge of generating similar models, resulting in similar prediction performances. This does not contribute to the predictive model at the output level unless the network generates diverse models during the training process. One way to overcome this challenge and build diverse models is to change the learning rate during the learning process. Thus, to this end, we exploited the Cyclic Cosine Annealing Schedule (CCAS) [17], that splits the training process into *N* cycles, each starting with a large learning rate and relatively rapidly decreasing to the minimum value before increasing quickly again. Equation (13) shows the learning rate over the iterations.

(13) $$\alpha \left(t\right)=\frac{\alpha_{0}}{2}\left(cos\left(\frac{\pi \;mod(t-1,\lceil T/n\rceil )}{\left\lceil T/N\right\rceil }\right)+1\right)$$

where, α denotes the learning rate at iteration *t*, $\alpha_{0}$ is the initial learning rate (maximum rate), and *T* refers to the total number of the training iterations. Thus, the function anneals the learning rate from $\alpha_{0}$ to f(T/C) which is ≈0 over the course of a cycle [72]. Value *N* is the number of cycles in which the network is trained throughout the training process, and in which the best weights at the end of each cycle are saved as the snapshot of the model. This results in *N* model snapshots. In fact, in this process, the learning rate is raised periodically, reinforcing the model’s convergence at the global minimum, rather than at the local minimum, over the snapshot ensemble. This leads to elimination of the need to manually find the optimal maximum learning rate. The overall snapshot process is illustrated in Figure 6, where at the end of each iteration the model with the maximum performance is saved as the snapshot. All the models are then used to predict the outcome in the first phase. The use of CCAS does not ensure diversity of the produced models, due to the stochastic nature of the algorithm. Therefore, the diversity of the snapshot models were carefully studied by computing and investigating any dissimilarities between prediction outputs. In short, in this step, we generated multiple diverse deep neural networks to build an ensemble of predictive models. This ensemble of models was then used to construct meta-learning (in the second phase) for the final breakdown prediction.

#### 4.4.2. Training the Network

Table 1 shows the construction of the network in the first phase, wherein we trained the ANN network by incrementally creating a Sequential model. The network parameters were initialized using “He” initializer [73] (shown in Equation (14)), where G is the Gaussian probability distribution with a zero-centered and standard deviation of $\sqrt{\frac{2}{n_{l}}}$. In this initialization form, biases are initialized at 0 and $n_{l}$ is the number of inputs to that node in layer *l*.

(14) $$w_{l}=G\left(0,\sqrt{\frac{2}{n_{l}}}\right)$$

In order to use stochastic gradient descent to train the deep neural networks, rectified linear unit (ReLU) [74] as the–active function–was utilized to transfer the summed weighted input from the nodes into the output of the node.

(15) f(x)=max(o,x)

In Equation (15), f(x) is the ReLU function, where it outputs 0 for ∀x<0, while for positive inputs x≥0, it returns the same value f(x)=x. The Batch Normalization [75] technique is used to normalize the input of each layer to reduce the internal covariate shift problem leading to stabilizing the learning process. This is followed by a stochastic gradient descent technique called “Adam” optimization, which iteratively updates network weights during the training process [76].

We used a regularization method called “Dropout” to reduce the over-fitting issue. Basically, this led to the provision of different neural network architectures when training the networks. In the output layer, we utilized the Soft-max activation function–depicted in Equation (16)–for the probability distribution of the target variables.

(16) $$\sigma \left(z_{i}\right)=\frac{e^{z_{i}}}{\sum_{j=1}^{K}e^{z_{j}}}$$

where $\sigma (z_{i})$ are the elements of the input vector to the softmax function, K is the number of classes and $\sum_{j=1}^{K}e^{z_{j}}$ is the normalization term, which ensures all the output values of the function sum to 1 (each class probability is in the range (0, 1)). Finally, the output of Equation (16) is injected to the Argmax function–defined in Equation (17) to predict the hard label.

(17) $$hp_{r_{i}}=argmax\left(\sigma \left(z_{i}\right)\right)$$

where $hp_{r_{i}}$ carries the hard label prediction of the readout $r_{i}$ in the test set, expressing whether the usage corresponds to a claim/breakdown (1) or not (0).

#### 4.4.3. Stacking and Horizontal Ensemble

Each snapshot model provides two types of outputs; Soft prediction (Equation (16)) and Hard label (Equation (17)). The former returns two values: the predicted probability of the readout belonging to a vehicle not having a claim and the predicted probability indicating the usage corresponding to a claim/fault. The second output is the hard label representing whether the component is healthy (0), given the vehicle usage, or faulty (1). Accordingly, Equation (18) shows the numbers of the new features which are generated by the Snapshots models.

(18) $$feature_{new}=N_{Sn}\times \sum_{k=1}^{f}\left(C_{k}+1\right)$$

where $N_{S_{n}}$ refers to the snapshot numbers, and *f* is the number of features. $C_{k}$ points to the binary class of the vehicle status (healthy–0– or unhealthy–1–) which is summed by “1” as the hard-label of that prediction. Table 2 illustrates the snapshot and stacking horizontal ensembles calculations, which are described in detail as follow:

– $r_{1},\ldots ,r_{n}$: refers to the vehicle non-stationary readouts/samples, which are collected over time.
– $f_{1},\ldots ,f_{m}$: represents the LVD parameters characterizing a vehicle’s behavior in $r_{i}$.
– $P_{r_{1}\left(sp_{1}\right)}\ldots P_{r_{1}\left(sp_{m}\right)}$, indicates the output of the snapshot models–in the first phase–for the given readouts.
– $p_{c_{1}},p_{c_{2}}$, represents the predicted probability of class 0 (healthy) and class 1 (unhealthy), for the given readout $r_{i}$.
– $hp_{r_{i}}$ refers to the hard prediction of the given readout $r_{i}$ (0 or 1).

**Figure 7:**
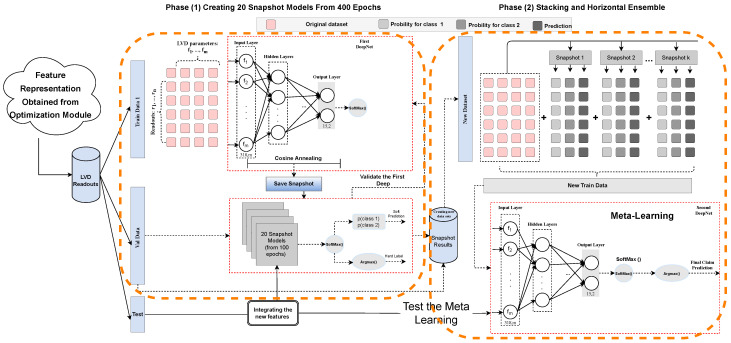
The schematic representation of the third module starts by partitioning the LVD data (injected from Module2) into three parts: training data, validation data, and test data. The training and validation are used in the first phase to generate the snapshot (SPs) models. In this process, CCSA is utilized to construct the snapshots where the function periodically decreases the learning rate from $\alpha_{0}$ to ≈0 in a cycle; in this study, it was every 20 snapshots (see Figure 6). Then, at the end of each cycle, the model with the maximum performance is saved as the snapshot. In the second phase, the output of the SPs is horizontally added to the original set (validation data) to make a new dataset. Accordingly, the new dataset is used to build the meta-learning (with the training part) for the final prediction. Finally, the test part is utilized (with the new dimensions) to validate the meta-learning performance.

The output of the soft predictions and the hard labels are horizontally combined with the original features (validation set, see Figure 7). This means that, by considering Equation (18), each snapshot model could add three extra parameters carrying knowledge of the previous model’s performance on that specific readout. The new data–as the new training set–is then passed to the second deep net to build the meta-learning to reduce the forecasting errors obtained from the previous phase. Indeed, meta-learning is built by stacking the outputs of the snapshots, which are diverse deep neural networks and able to capture the expected complex signal relations, as compared to learning algorithms with less capacity, see Tables 5 and 6. As is described above, CCAS is utilized to construct 20 different predictive deep network models in this stacking process. To obtain the final fault prediction, we injected the test set, which was held out, into the same generated snapshots to translate the test set’s structure to the new one with more dimensions, similar to the new training set (see Figure 3 left-bottom). Finally, the performance of the meta-learning is assessed by the test set. Table 1 (right side) describes the architecture of the second deep network, and Algorithm 1 shows the pseudo code of the whole process in this complex claim prediction.
**Algorithm 1** The proposed SSED approach, Module 1, 2 and 3**Input:** *R*▹readouts R=$\left\{r_{1},\ldots ,r_{n}\right\}$**Output:** Fp▹final claim prediction 1: Trd∈R▹training data 2: Vld∈R▹validation data 3: Tsd∈R▹test data 4: *S*▹Snapshot 5: Ls←{}▹list of snapshots 6: Lseg←{}▹list of segments 7: Linf←{}▹list of informative features 8: Dtr←{}▹new dataset for training 9: Dts←{}▹new dataset for test10: CCAS▹cosine annealing function (Equation (13))11: *N*▹number of cycles, (Equation (13))12: Deep1▹1st network to generate snapshots (Table 1-left side)13: Deep2▹2nd network for final prediction (Table 1-right side)14: Module 1 is started:15: R←label(R)▹label R, Equation (1)16: Lseg←Segment(R)▹partition R into four segments17: Module 2 is started:18: **for** 
$Sg_{i}\;\in \;Lseg$ 
**do**19:    **while** Crisnotmet **do**20:        $Linf\leftarrow \;Optimization(Sg_{i})$▹ select informative features21:    **end while**22: **end for**23: $R_{new}\;\leftarrow \;Union\left(Linf\right)$▹union the informative features24: 
Module 3-phase 1 is started:25: 
$Train\;(Deep1\;with\;CCAS|R_{new})$26: **while** 
i≤E 
**do**27:    **if** mod(i∖N)==0 **then**28:        Ls←S▹ save the snapshot in Ls29:    **end if**30: **end while**31: LoadLs▹load all snapshots32: **for** 
$S_{i}\;\in \;Ls$ 
**do**33:    $p=S_{i}.predict\left(Vld\right)$▹validate $S_{i}$ with validation set34: **end for**35: Module 3-phase 2 is started:36: Creating a new data set37: **for** 
$S_{i}\;\in \;Ls$ 
**do**38:    Dtr←stack(p,Vld)▹ stack the prediction to Vld39: **end for**40: **for** 
$S_{i}\;\in \;Ls$ 
**do**41:     Dts←stack(p,Tsd)▹ stack the prediction to Tsd42: **end for**43: Mm←Train(Deep2∣Dtr)▹building meta-learning44: Fp←Mm.predict(Dts)▹final prediction

## 5. Experimental Evaluation and Results

To facilitate the implementation of the approach, we recall the two research questions, introduced in Section 1, on which we based the evaluation of the proposed approach as follows:

### 5.1. RQ1—Feature Selection with Optimization Results

Bearing in mind RQ1, we constructed two evaluation tracks. Firstly, the proposed optimization approach (ELSGA) was evaluated over several generations and compared with the standard genetic algorithm (without the IC component). Secondly, the selected features (using ELSGA) were used to build models through a type of time-series k-fold cross validation called “walk-forward cross validation (WFCV)” [77] and compared with other ML, deep learning and ensemble based approaches.

The optimization process was carried out as feature selection by selecting the most informative parameters to predict a vehicle’s component failures (a component related to the turbocharger). We built a forecasting system in which a supervised ML algorithm examined the contribution of the parameters to the claims. Considering results from our previous study [18], in which vehicle usage changed over time, we segmented the data into different parts. This segmentation process was done by assuming that the distribution of the vehicle usage logged in the same season over the years was relatively similar with respect to the other seasons. Thus, in each segment there might be a set of parameters that had more impact on the failures, and, accordingly, more pertinent to the prediction of failure. We designed the optimization process to be implemented in each segment in order to identify the most informative features. However, in the second tier of the evaluation, the union of the selected features (from all segments) built the predictive models, and was compared with other algorithms.

Since there is no universal configuration for such a heuristic optimization algorithm that is considered to be the best setting, GA parameters, including population size, mutation rate, number of parents inside the mating pool, and number of elements to mutate, were selected in an optimization manner. This meant the implementation code was constructed in a parameterized fashion to find the best configuration of the GA settings. Table 3 lists the parameters that our optimization system tuned to obtain the best performance.

Table 4 describes the output of the IC at initiating the first population on the whole and segmented data. The values α and l1−norm were selected through the WFCV (as the optimal values) to train the elastic model.

The whole data-set with the lowest feature reduction (FR=0.29%) resulted in the highest number of predictors in the first population, with 410. Among the segments, 313 predictors were selected with coefficients larger than 0 in Seg4, which also indicated the lowest reduction proportion within the segments. Likewise, the IC initiated 80 predictors out of 577 as being decent parents for further generation in Seg1. The figures indicated that the IC delivered different numbers of predictors to GA operators–meaning that, in each segment/season, different parameters had different impacts on failure of further generations in each segment. It was observed the value of α in all cases was close to zero, which meant that the LS almost turned into the ridge method to initiate the first generation.

To evaluate the performance of the selected features, we formulated the objective function as a classification task, wherein the function seeks the optimal performance of the breakdowns forecasting, taking into account vehicle usage. Thus, given the vehicle usage with *n* readout samples and the selected predictors/features *m*, which were injected by GA operators, $D=\left(r_{i},t_{i}\right)\left(|D\right|=n,r_{i_{f1\ldots ,fm}},t_{i}\in \left(0,1\right))$ in each generation, the function predicted whether the usage led to a failure or not.

In this stage of the experiment, the data was partitioned into training and test data. More to the point, in each segment of the data “throughout the optimization process”, we took three months of vehicle operation, with one month from the previous season, into account to train the model, and the last month was used to test the model. The statistical information of the training and test data in each segment is listed as follows:

- Segment1 (Seg1) contained 86,768 readouts, where 52,060 were considered for training and 34,708 for testing the model.
- Segment2 (Seg2) contained 90,709 readouts, where 54,421 were considered for training and 36,288 for testing the model.
- Segment3 (Seg3) contained 61,364 readouts, where 38,045 were considered for training and 23,319 for testing the model.
- Segment4 (Seg4) contained 98,732 readouts, where 75,036 were considered for training and 23,696 for testing the model.

As is mentioned in the Segmentation section, in each segment, the readout time-series samples were placed in the time order, in order to use the past data to build the model, and future data to test the model. Since these data were cumulative, and the scale of the readouts differed from year to year, data normalization–using Equation (19)–was conducted to form the data on the same scale.

(19) $$normalize\_value=\frac{value-min}{max-min}$$

The three criteria, $tr_{1}$, $tr_{2}$ and $tr_{3}$, were set to 0.5, 0.95 and 100 to recall the IC or terminate the optimization process so as to provide the best predictors, respectively.

**Figure 8:**
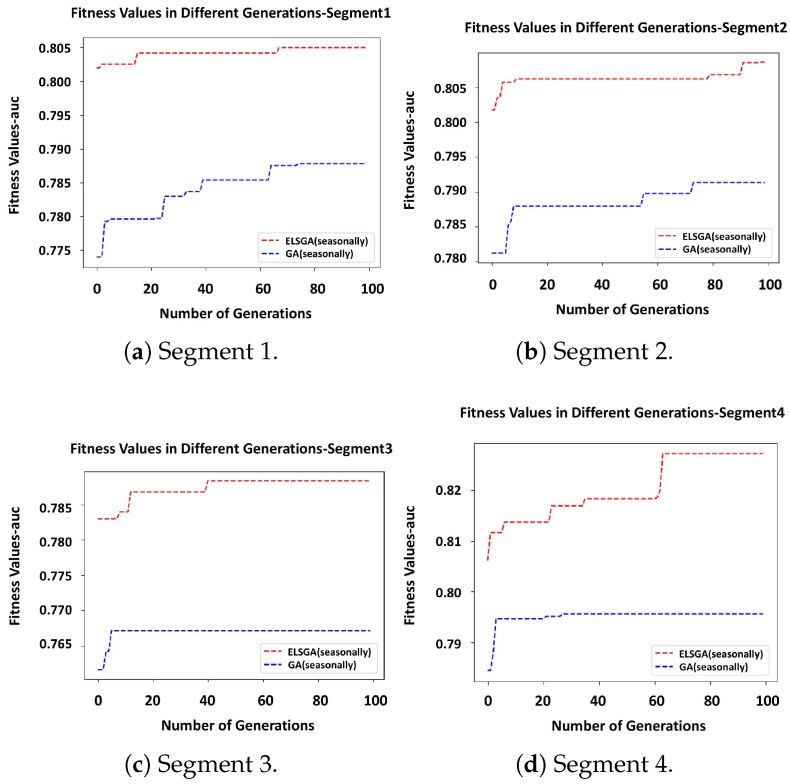
The performance of the proposed ELSGA and GA in each segment.

The plots in Figure 8 illustrate the superiority of the ELSGA approach in all segments compared with the GA approach without the IC component over the whole optimization process. We noticed a considerable jump in the performance provided by ELSGA that describes how well the IC adapted to the optimization process to generate decent individuals in the first population. The increase at the early stages assured that the whole optimization process could be terminated before the criterion $tr_{3}$ was met (maximum 100 generation). However, $tr_{2}\geq 95\%$ was not passed due to the complexity of the problem. ELSGA performed better by reporting around 80% AUC in all segments with 3% higher than GA over the process. It was also noticeable that the AUC value obtained by ELSGA in *Segment4* was higher among the other segments, with 82% AUC vs. 79% obtained by the basic optimization. The statistical assessment of the data revealed that *Segment4* contained vehicles which had more claims compared to the vehicles located in other segments in this highly unbalanced data. This might be the reason why our model performed better, since more data and positive samples were available to build the predictive model.

The overall results suggest the segmentation formulation is compatible and positively contributes to better claim prediction performance with optimization approaches, particularly the ELSGA approach.

In the second track of the evaluation, the output of the ELSGA was taken into consideration to build the model using the XGBoost classifier [68]. In this experiment, the selected predictors, due to ELSGA, were exploited to build the predictive models and to compare with models trained with several classifiers by injecting the data. In fact, multiple classifier (including deep learning and ensemble approaches) results were selected as the baseline to compare with ELSGA. The similar WFCV method was used to build and evaluate the models with various portions of the data in a time-series fashion (this decision was made since our data was time-series data and the folds could not be randomly selected by normal k-fold cross-validation).

Table 5 shows the comparison of our proposed ELSGA method and multiple classifiers. The figures obtained from different predictive models demonstrate how complex the task is to predict claims by taking vehicle usage into account. As can be seen, most of the classifiers performed poorly, providing low auc≈0.50 values. Practically, these results showed that most of the linear classifiers had no discriminatory capacity in regard to this complex problem, and could not map the usage to breakdowns. In contrast, deep learning models (such as CNN, LSTM and biLSTM) showed much better performance compared to the linear classifiers, by auc=0.70±2.

Among the examined predictive models, Boosting and Stacking performed close to the proposed approach by auc=0.75 and 0.74 vs. auc=0.76, respectively, when total data was considered. Concerning the segmentation, we can clearly observe that the proposed approach (Module2-ELSGA) significantly outperformed the other classifiers, except for Bagging. It is necessary to remark that, within deep learning approaches, CNN worked well on the last segment by providing auc=0.77. To go beyond this performance assessment, we applied a 5×2cv paired statistical *t*-test to evaluate how significant the results of the classifiers were. In almost all cases, the *p*-values were smaller than the critical value α=0.05, so this rejected the null hypothesis, by stating that there was a significant difference between the outcomes. The statistical tests showed that only the performance of the ensemble-based approaches were relatively close to that of the proposed approach in most cases. It is also fair to remark that the statistical test on the performance of the CNN model, in the last segment, indicated that the difference was not significant. It can be observed that in one case even Bagging performed slightly better than the proposed approach in Segment 3; however, the difference was not significant. In Segment 1, the figures indicate Stacking performed quite similarly to our proposed ELSGA approach by reporting auc=0.81.

In addition, we conducted an A/B test by comparing the performance of the models trained by the original predictors (MORGP) vs. the models built by the extracted predictors (MEXP). Both groups of predictors (original and extracted) were selected by the optimization approach. Indeed, we aimed to reveal the contribution of the extracted features and original features to predictive models.

**Figure 9:**
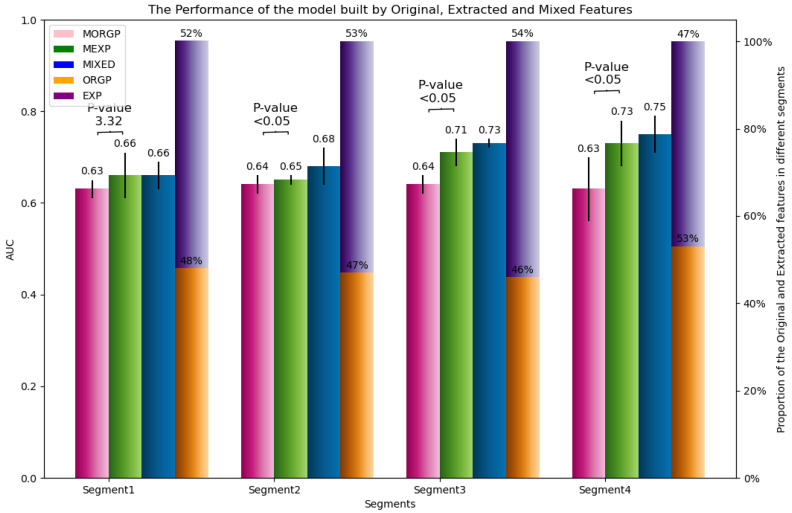
The outcome of the models built by different predictors. “ORGP” denotes the original predictors percentage, and “EXP” shows the proportion of the extracted predictors obtained after the optimization process.

From Figure 9, we can observe that more than 50% of the selected predictors (except in the last segment) were extracted from the extraction process, which translated to the importance of such predictors in the prediction task (Orange vs. purple bars). In fact, we considered this proportionate comparison mainly as a sanity check. However, to quantify the impact of the extracted features, several models were built through WFCV to construct and evaluate the models. The figures suggest the MEXP outperformed the MORGP by 3%, 1%, 7%, and 10% in segments one, two, three, and four, respectively (green vs. pink bars). The statistical tests also indicated how significant the differences were in segments two, three, and four by rejecting the null hypothesis (α is set to 0.05 as critical value). Although the test failed to reject the null hypothesis (by *p*-value = 3.32), and concluded the difference between the two models was not significant in the first segment (green and pink bars), the Extracted predictors (green) showed their valuable impacts by providing almost the same performances 0.66 with respect to the models trained by the mixed predictors (blue bar). The information extracted and used as extracted predictors in the models had greater, and more significant, impact on the predictive model’s performances, compared to the original predictors.

### 5.2. RQ2—Snapshot-Stacked Ensemble Results

To answer RQ2, we took the output of the optimization process as the input of the ensemble module. We aimed to build a general model that could be used to predict the claims over the year, given vehicle usage. Thus, identical features from all segments were combined and injected into the first deep network to build and generate several diverse snapshot models. In addition, each season, as one additional feature, was added to the identical features to support generalization.

**Figure 10:**
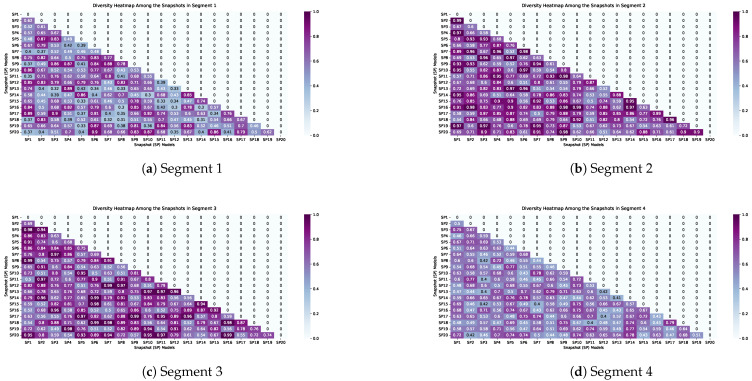
The disagreement of 20 snapshot models generated by cosine annealing in different segments. The heat-map shows how the generated snapshots were diverse in the first phase of Module2. In these plots, each cell under the orthogonal represents the disagreement between two snapshots–e.g., SP1 vs. SP2–. The more div (Equation (20)) value close to 1 the more diverse the models. From the heat-maps we observed that the snapshots in Segment 2 and Segment 3 were more diverse than in Segment 1 and Segment 4.

Given Equation (13), the number of cycles were set to N=20 to generate 20 different snapshot models over the 400 epochs. Of the data, 60% were considered to train the snapshots and 30% to be the validation set to obtain the hard labels and soft predictions. Accordingly, 10% of the data was held out to test the meta-learning model at the final stage (see Figure 7 for visualization purposes). To quantify the diversity in the generated models, we used a disagreement measure, defined in Equation (20). This metric calculated the relation between the number of times the base classifiers predicted the same label, in terms of the total number of instances [78].

(20) $$dis_{i,j}=\frac{1}{N}\sum_{k=1}^{N}fn(Sn_{i}\left(r_{k}\right)\neq Sn_{j}\left(r_{k}\right))$$

where $Sn_{i}\left(r_{k}\right)$ refers to the label assigned by the snapshot ($Sn_{i}$) to readout $r_{k}$. Value fn() is the function of truth predicate that counts the cases where $Sn_{i}$ was correct and the $Sn_{j}$ was wrong and vise-versa. Figure 10 illustrates the disagreement between the 20 snapshots. In the first phase, the hard labels predicted by the 20 snapshot models (here we took the average of the models’ performances) was compared with the performance of the same algorithms used in the second module (Module2) evaluation, depicted in Table 6. The AUC values obtained over the 5-fold WFCV showed that, in almost all cases, our approach was superior in the first phase (Module3 First phase–Snapshots–, see Table 6), compared to the other approaches. This was not the case when it came to comparing the ensemble-based and Module2 performances. The classification results from all segments (0.81 vs. 0.79 in Segment 1, 0.80 vs. 0.75 in Segment 2, 0.82 vs. 0.80 in Segment 3 and 0.82 vs. 0.78 in Segment 4) and union of the features (0.76 vs. 0.75) suggested the first module performed better with respect to the first deep net (first phase). A similar observation was obtained when the first phase was compared with other ensemble approaches, such as Bagging, Boosting, and Stacking. This meant we did not see any improvement from the snapshot models in the first deep neural networks.

This motivated us to construct meta-learning with the aim to learn from the errors received in the first phase and improve the prediction in the second phase, that being the final prediction. The results of the snapshot models (20×3) were horizontally added to the data-set (the validation set in the first phase) to be trained and tested again in the second phase. This resulted in 60 extra new features in the data set. This meant the data considered as the validation set in the first deep networks (first phase/layer) became a new data set by carrying 60 extra features (in the second phase). Accordingly, the test set (10% of the whole data), which was held out, was used to assess the model. It is necessary to remark that, in each phase, we trained the deep neural network separately in one training shot. This meant that, in the first phase, we constructed 20 models in one training process using CCAS, and, in the second phase, we built a meta-learning model through one training process.

The result of the second phase is shown in Table 6 (last row), which was compared with other approaches. The figures represented in the table confirm significant improvement due to the stacking and ensemble snapshots performances generated in the first phase. We almost obtained auc>0.80 in all experiments, with the exception of one in Segment 2 (auc=0.75). The meta-learning built by the snapshot models could learn the errors obtained from the previous training and prediction phases. This resulted in a general predictive model that could be potentially used for claim/fault prediction in all seasons. Indeed, the diversity of the snapshots, illustrated in Figure 10, highly supported the second phase to build a generalized meta-learning model for the final prediction. Taking into account the results obtained from the above experiments, the ensemble approaches performed well, compared with other classifiers, including linear and deep networks. However, the superiority of our approach in this complex problem was very evident. This led us to examine these approaches in a different context. Thus, we conducted the proposed approach with the other three ensemble approaches on several different datasets to assess the generality and to ascertain whether the SSED performed equally to, or better than, its performance in other application domains. This is an important consideration, since it assesses the generality of the approach to deal with data from different contexts (see Table 7 for the details information of the data sets).

The observations obtained from Table 8 show that SSED, in most cases, took the first rank, in terms of accuracy of performance, and outperformed other ensemble approaches. Individual comparison between SSED Vs. Bagging and Boosting indicated that the SSED almost consistently outperformed the two approaches on all datasets. However, this consistency was violated on dataset 7, where the figures show that Boosting provided slightly better results (%2). In contrast, the comparison between SSED vs. Stacking suggests that Staking had better generalization on the datasets. However, the statistical tests on the results (datasets 3, 7, 9, and 10) described the differences as not being statistically significant, when considering the critical value at α=0.05. Concerning the same comparison, we observed the value of the t-test on the figures obtained on datasets 1, 4, 6, 10, 11, where the SSED performed better, confirmed the differences were statistically significant, and concluded that SSED outperformed the Stacking approach.

## 6. Discussion and Conclusions and Future Work

This paper presents a two-phase snapshot-stacked ensemble approach to predict warranty claims/breakdowns in the automotive sector. The experimental evaluation, using the logged data from thousands of heavy-duty trucks, showed that the proposed approach could properly predict failures based on vehicle usage. Regarding the research questions introduced in Section 1, we evaluated the key aspects, which expressed our approach’s efficiency, effectiveness, stability and generality. Considering the first objective (RQ1), figures obtained in model performance experiments showed a significant difference between the optimization and other classifiers. The results in terms of *feature contribution* were auspicious. Our A/B test experiments, illustrated in Figure 9, explain how effectively the extracted features contributed to the predictive model. Statistical tests also indicated the improvement induced by the extracted features was significantly different from the original features. It was also observed that the model constructed by the extracted features in the first segment provided similar performances compared to the model trained by both the original and extracted predictors. Therefore, we answer RQ1 by stating that: *our proposed adapted heuristic approach outperforms the other approaches to extract the best representation of vehicle usage in order to build a complex ensemble deep network for final claim prediction*.

Concerning the second objective (RQ2), the results explained how meta-learning could learn from the errors received by the snapshot models in the first phase of the ensemble approach to predict warranty claims. Indeed, the results in the second phase confirmed the stability of the meta-learning performance over the course of the year, taking into account different distributions of vehicle usage. The SSED could produce a generic predictive model that performed well on data from different seasons for claim predictions but was also promising for other less complex datasets comparable to the performance of Stacking. Therefore, we answer RQ2 by stating that: *the snapshot-stacked ensemble deep neural networks approach can successfully map usage to claims and outperforms the other compared algorithms. The figures obtained in the last phase also showed the potential in building a general forecasting model for this complex claim prediction problem. In addition, the results obtained on various datasets confirmed this claim*.

The findings of this work also reveal limitations of the proposed approach, which suggest new directions for future research. The first limitation pertains to the issue of modeling vehicle usage by taking into account various contexts. In this work, our concerns were time and vehicle usage to formulate the claim prediction. However, we did not include other contexts, such as location (where the vehicles were driven), which highly affect usage and predictive models. This limitation strongly motivates us to extend our approach by incorporating context knowledge (e.g., location) for a better mapping between usage and breakdowns. An interesting extension of our work could focus on vehicle profiling. We could utilize segmentation to model vehicles’ behaviors in different contexts, leading to identification of good and bad driving styles as an important factor in vehicle performance. Thus, in this matter, driver behavior as an extra knowledge input could be used to characterize the style of vehicle usage in different contexts. Regarding contexts, we hypothesize that climate could impact snapshot diversity. This could also explain the dissimilarities shown in the results, which could be subject to further investigation. 

## Figures and Tables

**Table 1:** The architecture of the first deep networks–deep1–(left side) and the second deep network (right side). The values of the model hyper-parameters were selected experimentally in a systematic fashion.

Layers (Type)	Output Shape	# of Parameters	Layers (Type)	Output Shape	# of Parameters
Sequential()	–		Sequential()	–	
batch00 (BatchNormalization)	(None, 160)	640	batch00 (BatchNormalization)	(None, 214)	856
layer00 (Dense)	(None, 160)	25,760	layer00 (Dense)	(None, 214)	46,010
batch10 (BatchNormalization)	(None, 160)	640	batch10 (BatchNormalization)	(None, 214)	856
layer10 (Dense)	(None, 40)	6440	layer10 (Dense)	(None, 53)	11,395
batch20 (BatchNormalization)	(None, 40)	160	batch20 (BatchNormalization)	(None, 53)	212
layer20 (Dense)	(None, 13)	533	layer20 (Dense)	(None, 17)	918
batch30 (BatchNormalization)	(None, 13)	52	batch30 (BatchNormalization)	(None, 17)	68
dropout (Dropout)	(None, 13)	0	dropout (Dropout)	(None, 17)	0
layer30 (Dense)	(None, 8)	112	layer30 (Dense)	(None, 10)	180
batch40 (BatchNormalization)	(None, 8)	32	batch40 (BatchNormalization)	(None, 10)	40
layer40 (Dense)–softmax()	(None, 2)	18	layer40 (Dense)	(None, 2)	22
Total parameters:	34,387		Total parameters:	60,557	
Trainable parameters:	33,625		Trainable parameters:	59,541	
Non-trainable parameters:	762		Non-trainable parameters:	1,016	

**Table 2:** Illustration of the snapshot and stacked ensemble output.

Readouts	Original Features			Snapshot Models’ Predictions		
	$f_{1}$	…	$f_{m}$	$P_{r_{1}\left(sp_{1}\right)}=\left(p_{c1},p_{c2},hp_{r}\right)$	…	$P_{r_{1}\left(sp_{m}\right)}=\left(p_{c1},p_{c2},hp_{r}\right)$
$r_{1}$	$r_{1_{f_{1}}}$	…	$r_{1_{f_{m}}}$			
$r_{2}$	.	…				.
.	.	…				.
.	.	…				.
$r_{n}$	$r_{n_{f_{1}}}$	…	$r_{n_{f_{m}}}$	$P_{r_{n}\left(sp_{1}\right)}=\left(p_{c1},p_{c2},hp_{r_{n}}\right)$	…	$P_{r_{n}\left(sp_{m}\right)}=\left(p_{c1},p_{c2},hp_{r}\right)$

**Table 3:** The tuned parameters in the proposed optimization approach.

	Tested Parameters	Best Parameter
# of Generation	—	**100**
# of items to mutate	1–10	**3**
# of parents in mating pool	2, 4, 6, 8	**4**
Population size	6, 8, 10, 12, 14	**10**

**Table 4:** The Output of the Initialization Component.

	α	L1-Norm	Reduction %	# of Decent Predictors
Total Data	7.1 × 10${}^{-7}$	0.050	29.79%	410
Seg1	1.1 × 10${}^{-5}$	0.99	86.3%	80
Seg2	1.4 × 10${}^{-5}$	0.70	82.3%	103
Seg3	2.2 × 10${}^{+5}$	0.50	77.5%	131
Seg4	3 × 10${}^{-5}$	0.10	46.4%	313

**Table 5:** The comparison between Module2 (ELSGA (XGB)) and different approaches (standard machine learning, Gaussian Process (GP), deep neural networks, and ensemble-based approaches, such as Bagging, Boosting and Stacking approaches) for RQ1. We used a 5×2cv paired *t*-test to examine how significant the differences were between the performances of the two models. This meant that every time we compared only two models, one ELSGA vs. one classifier.

Classifiers	All Data		Segment1		Segment2		Segment3		Segment4	
	auc	*p*	auc	*p*	auc	*p*	auc	*p*	auc	*p*
**Logreg**	0.50±0.00	<0.05	0.50±0.00	<0.05	0.50±0.00	<0.05	0.50±0.00	<0.05	0.50±0.00	<0.05
**Kneig**	0.52±0.02	<0.05	0.544±0.01	<0.05	0.52±0.01	<0.05	0.54±0.04	<0.05	0.52±0.02	<0.05
**SGD**	0.50±0.00	<0.05	0.50±0.00	<0.05	0.50±0.00	<0.05	0.50±0.00	<0.05	0.50±0.00	<0.05
**QdA**	0.53±0.02	<0.05	0.50±0.00	<0.05	0.50±0.00	<0.05	0.50±0.00	<0.05	0.50±0.00	<0.05
**SVM**	0.55±0.05	<0.05	0.56±0.02	<0.05	0.53±0.03	<0.05	0.57±0.04	<0.05	0.57±0.06	<0.05
**DT**	0.54±0.01	<0.05	0.58±0.01	<0.05	0.59±0.02	<0.05	0.58±0.02	<0.05	0.60±0.03	<0.05
**Ridge**	0.71±0.07	<0.05	0.73±0.04	<0.05	0.70±0.06	<0.05	0.71±0.05	<0.05	0.71±0.07	<0.05
**SVC**	0.52±0.01	<0.05	0.52±0.01	<0.05	0.51±0.001	<0.05	0.51±0.001	<0.05	0.53±0.02	<0.05
**GP**	0.50±0.00	<0.05	0.50±0.00	<0.05	0.50±0.00	<0.05	0.50±0.00	<0.00	0.50±0.00	<0.05
**CNN**	0.65±0.08	<0.05	0.67±0.07	<0.05	0.70±0.06	<0.05	0.71±0.041	<0.05	0.77±0.11	0.14
**LSTM**	0.69±0.05	0.2	0.74±0.02	<0.05	0.71±0.06	<0.05	0.71±0.06	<0.05	0.72±0.05	<0.05
**biLSTM**	0.70±0.05	0.2	0.74±0.03	<0.05	0.70±0.07	<0.05	0.72±0.05	<0.05	0.72±0.10	<0.05
**Blending**	0.58±0.01	<0.05	0.58±0.004	<0.05	0.59±0.03	<0.05	0.58±0.01	<0.05	0.58±0.01	<0.05
**Bagging**	0.71±0.06	<0.05	0.80±0.04	0.24	0.78±0.06	0.30	0.81±0.06	**0.41**	0.79±0.09	0.35
**Boosting**	0.75±0.05	0.38	0.75±0.05	<0.05	0.76±0.06	0.19	0.76±0.04	0.21	0.75±0.09	0.15
**Stacking**	0.74±0.14	<0.05	**0.81** ± **0.01**	0.16	0.79±0.06	0.6	0.78±0.04	<0.05	0.81±0.08	0.3
**Module2 ELSGA (XGB)**	0.76±0.06		0.81±0.04		0.80±0.07		0.80±0.07		0.82±0.09	

**Table 6:** The comparison between Module 3 (SSED)-first phase, Module3 (SSED)-second phase and different approaches (standard machine learning, Gaussian Process (GP), deep neural networks, and ensemble based approaches, such as Bagging, Boosting and Stacking approaches) for RQ2. We used 5×2cv paired *t*-test to examine how significant the differences were between the performances of the approaches. In this statistical test, only “Module3 (SSED)-second phase” was compared with other algorithm performances. Every time we compared only two models; e.g., Module3 (SSED)–second phase vs. Boosting, or Module3 (SSED)–second phase vs. Module3 (SSED)–first phase, or Module2–ELSGA (XGB).

Classifiers	Union of the Features		Segment1		Segment2		Segment3		Segment4	
	auc	*p*	auc	*p*	auc	*p*	auc	*p*	auc	*p*
**Logreg**	0.50±0.00	<0.05	0.50±0.00	<0.05	0.50±0.00	<0.05	0.50±0.00	<0.05	0.50±0.00	<0.05
**Kneig**	0.52±0.02	<0.05	0.544±0.01	<0.05	0.52±0.01	<0.05	0.54±0.04	<0.05	0.52±0.02	<0.05
**SGD**	0.50±0.00	<0.05	0.50±0.00	<0.05	0.50±0.00	<0.05	0.50±0.00	<0.05	0.50±0.00	<0.05
**QdA**	0.53±0.02	<0.05	0.50±0.00	<0.05	0.50±0.00	<0.05	0.50±0.00	<0.05	0.50±0.00	<0.05
**SVM**	0.55±0.05	<0.05	0.56±0.02	<0.05	0.53±0.03	<0.05	0.57±0.04	<0.05	0.57±0.06	<0.05
**DT**	0.54±0.01	<0.05	0.58±0.01	<0.05	0.59±0.02	<0.05	0.58±0.02	<0.05	0.60±0.03	<0.05
**Ridge**	0.71±0.07	<0.05	0.73±0.04	<0.05	0.70±0.06	<0.05	0.71±0.05	<0.05	0.71±0.07	<0.05
**SVC**	0.52±0.01	<0.05	0.52±0.01	<0.05	0.51±0.001	<0.05	0.51±0.001	<0.05	0.53±0.02	<0.05
**GP**	0.50±0.00	<0.05	0.50±0.00	<0.05	0.50±0.00	<0.05	0.50±0.00	<0.00	0.50±0.00	<0.05
**CNN**	0.65±0.08	<0.05	0.67±0.07	<0.05	0.70±0.06	0.12	0.71±0.041	<0.05	0.77±0.11	0.2
**LSTM**	0.69±0.05	0.06	0.74±0.02	<0.05	0.71±0.06	0.19	0.71±0.06	<0.05	0.72±0.05	<0.05
**biLSTM**	0.70±0.05	0.2	0.74±0.03	<0.05	0.70±0.07	<0.05	0.72±0.05	<0.05	0.72±0.10	<0.05
**Blending**	0.58±0.01	<0.05	0.58±0.004	<0.05	0.59±0.03	<0.05	0.58±0.01	<0.05	0.58±0.01	<0.05
**Bagging**	0.71±0.06	0.10	0.80±0.04	0.10	0.78±0.06	0.25	0.81±0.06	0.37	0.79±0.09	0.25
**Boosting**	0.75±0.05	0.21	0.75±0.05	<0.05	0.76±0.06	0.43	0.76±0.04	0.09	0.75±0.09	0.09
**Stacking**	0.74±0.14	<0.05	0.81±0.01	<0.05	0.79±0.06	0.14	0.78±0.04	<0.05	0.81±0.08	<0.05
**Module2 ELSGA (XGB)**	0.76±0.06	0.28	0.81±0.04	0.26	0.80±0.07	0.12	0.80±0.07	0.30	0.82±0.09	0.38
**Module3 SSED First phase (Snapshots)**	0.75±0.04	0.21	0.79±0.03	0.05	0.75±0.07	0.46	0.80±0.05	0.30	0.78±0.10	0.20
Module3 SSED Second phase (Stacked Ensemble)	0.80±0.11	—	0.83±0.04	—	0.75±0.04	—	0.82±0.07	—	0.84±0.08	—

**Table 7:** The detailed information of the datasets.

#	Database	# of Samples	# of Features	# of Classes
1	VBS-fault	8712	4	2
2	Car insurance claim	10,302	27	2
3	Covtype	581,012	55	3
4	Cmiyc	27,297	22	2
5	Kdd	494,021	42	17
6	Building permit	198,900	43	8
7	Diabetes	768	9	2
8	Mnist	9271	787	10
9	Musk	6598	168	2
10	Parkinson	756	757	2
11	Spam	4400	59	2
12	Waveform	5000	41	3
13	Australia	142,193	24	2

**Table 8:** The comparison between SSED and three ensemble approaches on various datasets. The statistical *t*-test was applied to the performances of the approaches. Each time, SSED and one approach (e.g., Bagging) was compared, and the *p*-value reported.

#	Datasets	Accuracy						
		SSED	Bagging	*p*-Value	Boosting	*p*-Value	Stacking	*p*-Value
1	VBS fault	**0.95** ± **0.02**	0.93±0.01	<0.05	0.75±0.35	0.14	0.93±0.01	<0.05
2	Car insurance claim	0.77±0.01	0.73±0.006	0.069	0.74±0.004	0.27	0.73±0.001	0.067
3	Covtype	0.81±0.1	0.68±0.009	<0.05	0.63±0.002	<0.05	0.84±0.005	0.32
4	Cmiyc	0.98±0.01	0.91±0.01	<0.05	0.93±0.006	<0.05	0.91±0.004	<0.05
5	Kdd	0.80±0.23	0.36±0.34	<0.05	0.78±0.001	0.480	0.62±0.43	0.288
6	Building permit	0.93±0.02	0.89±0.003	<0.05	0.94±0.004	<0.05	0.91±0.002	<0.05
7	Diabetes	0.73±0.11	0.75±0.03	0.35	0.74±0.04	0.41	0.75±0.03	0.35
8	Mnist	0.93±0.04	0.85±0.004	<0.05	0.86±0.008	<0.05	0.99±0.004	<0.05
9	Musk	0.97±0.01	0.98±0.002	0.28	0.84±0.3	0.19	0.99±0.01	0.282
10	Parkinson	0.94±0.02	0.73±0.05	<0.05	0.82±0.04	<0.05	0.77±0.02	<0.05
11	Spam	0.87±0.15	0.82±0.21	0.46	0.91±0.17	0.35	0.98±0.003	0.10
12	Waveform	0.88±0.02	0.86±0.003	0.34	0.82±0.01	<0.05	0.86±0.01	0.23
13	Australia	0.99±0.003	0.81±0.003	<0.05	0.99±0.007	0.42	0.99±0.01	0.29

## Data Availability

Data sharing not applicable.
